# Energy dependence of the transverse momentum distributions of charged particles in pp collisions measured by ALICE

**DOI:** 10.1140/epjc/s10052-013-2662-9

**Published:** 2013-12-06

**Authors:** The ALICE Collaboration, B. Abelev, J. Adam, D. Adamová, A. M. Adare, M. M. Aggarwal, G. Aglieri Rinella, M. Agnello, A. G. Agocs, A. Agostinelli, Z. Ahammed, N. Ahmad, A. Ahmad Masoodi, I. Ahmed, S. A. Ahn, S. U. Ahn, I. Aimo, S. Aiola, M. Ajaz, A. Akindinov, D. Aleksandrov, B. Alessandro, D. Alexandre, A. Alici, A. Alkin, J. Alme, T. Alt, V. Altini, S. Altinpinar, I. Altsybeev, C. Alves Garcia Prado, C. Andrei, A. Andronic, V. Anguelov, J. Anielski, T. Antičić, F. Antinori, P. Antonioli, L. Aphecetche, H. Appelshäuser, N. Arbor, S. Arcelli, N. Armesto, R. Arnaldi, T. Aronsson, I. C. Arsene, M. Arslandok, A. Augustinus, R. Averbeck, T. C. Awes, J. Äystö, M. D. Azmi, M. Bach, A. Badalà, Y. W. Baek, R. Bailhache, R. Bala, A. Baldisseri, F. Baltasar Dos Santos Pedrosa, J. Bán, R. C. Baral, R. Barbera, F. Barile, G. G. Barnaföldi, L. S. Barnby, V. Barret, J. Bartke, M. Basile, N. Bastid, S. Basu, B. Bathen, G. Batigne, B. Batyunya, P. C. Batzing, C. Baumann, I. G. Bearden, H. Beck, C. Bedda, N. K. Behera, I. Belikov, F. Bellini, R. Bellwied, E. Belmont-Moreno, G. Bencedi, S. Beole, I. Berceanu, A. Bercuci, Y. Berdnikov, D. Berenyi, A. A. E. Bergognon, R. A. Bertens, D. Berzano, L. Betev, A. Bhasin, A. K. Bhati, J. Bhom, L. Bianchi, N. Bianchi, C. Bianchin, J. Bielčík, J. Bielčíková, A. Bilandzic, S. Bjelogrlic, F. Blanco, F. Blanco, D. Blau, C. Blume, F. Bock, A. Bogdanov, H. Bøggild, M. Bogolyubsky, L. Boldizsár, M. Bombara, J. Book, H. Borel, A. Borissov, J. Bornschein, M. Botje, E. Botta, S. Böttger, E. Braidot, P. Braun-Munzinger, M. Bregant, T. Breitner, T. A. Broker, T. A. Browning, M. Broz, R. Brun, E. Bruna, G. E. Bruno, D. Budnikov, H. Buesching, S. Bufalino, P. Buncic, O. Busch, Z. Buthelezi, D. Caffarri, X. Cai, H. Caines, A. Caliva, E. Calvo Villar, P. Camerini, V. Canoa Roman, G. Cara Romeo, F. Carena, W. Carena, F. Carminati, A. Casanova Díaz, J. Castillo Castellanos, E. A. R. Casula, V. Catanescu, C. Cavicchioli, C. Ceballos Sanchez, J. Cepila, P. Cerello, B. Chang, S. Chapeland, J. L. Charvet, S. Chattopadhyay, S. Chattopadhyay, M. Cherney, C. Cheshkov, B. Cheynis, V. Chibante Barroso, D. D. Chinellato, P. Chochula, M. Chojnacki, S. Choudhury, P. Christakoglou, C. H. Christensen, P. Christiansen, T. Chujo, S. U. Chung, C. Cicalo, L. Cifarelli, F. Cindolo, J. Cleymans, F. Colamaria, D. Colella, A. Collu, M. Colocci, G. Conesa Balbastre, Z. Conesa del Valle, M. E. Connors, G. Contin, J. G. Contreras, T. M. Cormier, Y. Corrales Morales, P. Cortese, I. Cortés Maldonado, M. R. Cosentino, F. Costa, P. Crochet, R. Cruz Albino, E. Cuautle, L. Cunqueiro, A. Dainese, R. Dang, A. Danu, K. Das, D. Das, I. Das, A. Dash, S. Dash, S. De, H. Delagrange, A. Deloff, E. Dénes, A. Deppman, G. O. V. de Barros, A. De Caro, G. de Cataldo, J. de Cuveland, A. De Falco, D. De Gruttola, N. De Marco, S. De Pasquale, R. de Rooij, M. A. Diaz Corchero, T. Dietel, R. Divià, D. Di Bari, C. Di Giglio, S. Di Liberto, A. Di Mauro, P. Di Nezza, Ø. Djuvsland, A. Dobrin, T. Dobrowolski, B. Dönigus, O. Dordic, A. K. Dubey, A. Dubla, L. Ducroux, P. Dupieux, A. K. Dutta Majumdar, G. D Erasmo, D. Elia, D. Emschermann, H. Engel, B. Erazmus, H. A. Erdal, D. Eschweiler, B. Espagnon, M. Estienne, S. Esumi, D. Evans, S. Evdokimov, G. Eyyubova, D. Fabris, J. Faivre, D. Falchieri, A. Fantoni, M. Fasel, D. Fehlker, L. Feldkamp, D. Felea, A. Feliciello, G. Feofilov, A. Fernández Téllez, E. G. Ferreiro, A. Ferretti, A. Festanti, J. Figiel, M. A. S. Figueredo, S. Filchagin, D. Finogeev, F. M. Fionda, E. M. Fiore, E. Floratos, M. Floris, S. Foertsch, P. Foka, S. Fokin, E. Fragiacomo, A. Francescon, U. Frankenfeld, U. Fuchs, C. Furget, M. Fusco Girard, J. J. Gaardhøje, M. Gagliardi, A. Gago, M. Gallio, D. R. Gangadharan, P. Ganoti, C. Garabatos, E. Garcia-Solis, C. Gargiulo, I. Garishvili, J. Gerhard, M. Germain, A. Gheata, M. Gheata, B. Ghidini, P. Ghosh, P. Gianotti, P. Giubellino, E. Gladysz-Dziadus, P. Glässel, L. Goerlich, R. Gomez, P. González-Zamora, S. Gorbunov, S. Gotovac, L. K. Graczykowski, R. Grajcarek, A. Grelli, C. Grigoras, A. Grigoras, V. Grigoriev, A. Grigoryan, S. Grigoryan, B. Grinyov, N. Grion, J. F. Grosse-Oetringhaus, J.-Y. Grossiord, R. Grosso, F. Guber, R. Guernane, B. Guerzoni, M. Guilbaud, K. Gulbrandsen, H. Gulkanyan, T. Gunji, A. Gupta, R. Gupta, K. H. Khan, R. Haake, Ø. Haaland, C. Hadjidakis, M. Haiduc, H. Hamagaki, G. Hamar, L. D. Hanratty, A. Hansen, J. W. Harris, A. Harton, D. Hatzifotiadou, S. Hayashi, A. Hayrapetyan, S. T. Heckel, M. Heide, H. Helstrup, A. Herghelegiu, G. Herrera Corral, N. Herrmann, B. A. Hess, K. F. Hetland, B. Hicks, B. Hippolyte, Y. Hori, P. Hristov, I. Hřivnáčová, M. Huang, T. J. Humanic, D. Hutter, D. S. Hwang, R. Ichou, R. Ilkaev, I. Ilkiv, M. Inaba, E. Incani, G. M. Innocenti, C. Ionita, M. Ippolitov, M. Irfan, V. Ivanov, M. Ivanov, O. Ivanytskyi, A. Jachołkowski, C. Jahnke, H. J. Jang, M. A. Janik, P. H. S. Y. Jayarathna, S. Jena, R. T. Jimenez Bustamante, P. G. Jones, H. Jung, A. Jusko, S. Kalcher, P. Kaliňák, T. Kalliokoski, A. Kalweit, J. H. Kang, V. Kaplin, S. Kar, A. Karasu Uysal, O. Karavichev, T. Karavicheva, E. Karpechev, A. Kazantsev, U. Kebschull, R. Keidel, B. Ketzer, S. A. Khan, M. M. Khan, P. Khan, A. Khanzadeev, Y. Kharlov, B. Kileng, S. Kim, D. W. Kim, D. J. Kim, B. Kim, T. Kim, M. Kim, M. Kim, J. S. Kim, S. Kirsch, I. Kisel, S. Kiselev, A. Kisiel, G. Kiss, J. L. Klay, J. Klein, C. Klein-Bösing, A. Kluge, M. L. Knichel, A. G. Knospe, M. K. Köhler, T. Kollegger, A. Kolojvari, V. Kondratiev, N. Kondratyeva, A. Konevskikh, V. Kovalenko, M. Kowalski, S. Kox, G. Koyithatta Meethaleveedu, J. Kral, I. Králik, F. Kramer, A. Kravčáková, M. Krelina, M. Kretz, M. Krivda, F. Krizek, M. Krus, E. Kryshen, M. Krzewicki, V. Kucera, Y. Kucheriaev, T. Kugathasan, C. Kuhn, P. G. Kuijer, I. Kulakov, J. Kumar, P. Kurashvili, A. B. Kurepin, A. Kurepin, A. Kuryakin, S. Kushpil, V. Kushpil, M. J. Kweon, Y. Kwon, P. Ladrón de Guevara, C. Lagana Fernandes, I. Lakomov, R. Langoy, C. Lara, A. Lardeux, S. L. La Pointe, P. La Rocca, R. Lea, M. Lechman, S. C. Lee, G. R. Lee, I. Legrand, J. Lehnert, R. C. Lemmon, M. Lenhardt, V. Lenti, I. León Monzón, P. Lévai, S. Li, J. Lien, R. Lietava, S. Lindal, V. Lindenstruth, C. Lippmann, M. A. Lisa, H. M. Ljunggren, D. F. Lodato, P. I. Loenne, V. R. Loggins, V. Loginov, D. Lohner, C. Loizides, K. K. Loo, X. Lopez, E. López Torres, G. Løvhøiden, X.-G. Lu, P. Luettig, M. Lunardon, J. Luo, G. Luparello, C. Luzzi, P. M. Jacobs, R. Ma, A. Maevskaya, M. Mager, D. P. Mahapatra, A. Maire, M. Malaev, I. Maldonado Cervantes, L. Malinina, D. Mal’Kevich, P. Malzacher, A. Mamonov, L. Manceau, V. Manko, F. Manso, V. Manzari, M. Marchisone, J. Mareš, G. V. Margagliotti, A. Margotti, A. Marín, C. Markert, M. Marquard, I. Martashvili, N. A. Martin, P. Martinengo, M. I. Martínez, G. Martínez García, J. Martin Blanco, Y. Martynov, A. Mas, S. Masciocchi, M. Masera, A. Masoni, L. Massacrier, A. Mastroserio, A. Matyja, J. Mazer, R. Mazumder, M. A. Mazzoni, F. Meddi, A. Menchaca-Rocha, J. Mercado Pérez, M. Meres, Y. Miake, K. Mikhaylov, L. Milano, J. Milosevic, A. Mischke, A. N. Mishra, D. Miśkowiec, C. Mitu, J. Mlynarz, B. Mohanty, L. Molnar, L. Montaño Zetina, M. Monteno, E. Montes, T. Moon, M. Morando, D. A. Moreira De Godoy, S. Moretto, A. Morreale, A. Morsch, V. Muccifora, E. Mudnic, S. Muhuri, M. Mukherjee, H. Müller, M. G. Munhoz, S. Murray, L. Musa, B. K. Nandi, R. Nania, E. Nappi, C. Nattrass, T. K. Nayak, S. Nazarenko, A. Nedosekin, M. Nicassio, M. Niculescu, B. S. Nielsen, S. Nikolaev, S. Nikulin, V. Nikulin, B. S. Nilsen, M. S. Nilsson, F. Noferini, P. Nomokonov, G. Nooren, A. Nyanin, A. Nyatha, J. Nystrand, H. Oeschler, S. K. Oh, S. Oh, L. Olah, J. Oleniacz, A. C. Oliveira Da Silva, J. Onderwaater, C. Oppedisano, A. Ortiz Velasquez, A. Oskarsson, J. Otwinowski, K. Oyama, Y. Pachmayer, M. Pachr, P. Pagano, G. Paić, F. Painke, C. Pajares, S. K. Pal, A. Palaha, A. Palmeri, V. Papikyan, G. S. Pappalardo, W. J. Park, A. Passfeld, D. I. Patalakha, V. Paticchio, B. Paul, T. Pawlak, T. Peitzmann, H. Pereira Da Costa, E. Pereira De Oliveira Filho, D. Peresunko, C. E. Pérez Lara, D. Perrino, W. Peryt, A. Pesci, Y. Pestov, V. Petráček, M. Petran, M. Petris, P. Petrov, M. Petrovici, C. Petta, S. Piano, M. Pikna, P. Pillot, O. Pinazza, L. Pinsky, N. Pitz, D. B. Piyarathna, M. Planinic, M. Płoskoń, J. Pluta, S. Pochybova, P. L. M. Podesta-Lerma, M. G. Poghosyan, B. Polichtchouk, N. Poljak, A. Pop, S. Porteboeuf-Houssais, V. Pospíšil, B. Potukuchi, S. K. Prasad, R. Preghenella, F. Prino, C. A. Pruneau, I. Pshenichnov, G. Puddu, V. Punin, J. Putschke, H. Qvigstad, A. Rachevski, A. Rademakers, J. Rak, A. Rakotozafindrabe, L. Ramello, S. Raniwala, R. Raniwala, S. S. Räsänen, B. T. Rascanu, D. Rathee, W. Rauch, A. W. Rauf, V. Razazi, K. F. Read, J. S. Real, K. Redlich, R. J. Reed, A. Rehman, P. Reichelt, M. Reicher, F. Reidt, R. Renfordt, A. R. Reolon, A. Reshetin, F. Rettig, J.-P. Revol, K. Reygers, L. Riccati, R. A. Ricci, T. Richert, M. Richter, P. Riedler, W. Riegler, F. Riggi, A. Rivetti, M. Rodríguez Cahuantzi, A. Rodriguez Manso, K. Røed, E. Rogochaya, S. Rohni, D. Rohr, D. Röhrich, R. Romita, F. Ronchetti, P. Rosnet, S. Rossegger, A. Rossi, P. Roy, C. Roy, A. J. Rubio Montero, R. Rui, R. Russo, E. Ryabinkin, A. Rybicki, S. Sadovsky, K. Šafařík, R. Sahoo, P. K. Sahu, J. Saini, H. Sakaguchi, S. Sakai, D. Sakata, C. A. Salgado, J. Salzwedel, S. Sambyal, V. Samsonov, X. Sanchez Castro, L. Šándor, A. Sandoval, M. Sano, G. Santagati, R. Santoro, D. Sarkar, E. Scapparone, F. Scarlassara, R. P. Scharenberg, C. Schiaua, R. Schicker, C. Schmidt, H. R. Schmidt, S. Schuchmann, J. Schukraft, M. Schulc, T. Schuster, Y. Schutz, K. Schwarz, K. Schweda, G. Scioli, E. Scomparin, R. Scott, P. A. Scott, G. Segato, I. Selyuzhenkov, J. Seo, S. Serci, E. Serradilla, A. Sevcenco, A. Shabetai, G. Shabratova, R. Shahoyan, S. Sharma, N. Sharma, K. Shigaki, K. Shtejer, Y. Sibiriak, S. Siddhanta, T. Siemiarczuk, D. Silvermyr, C. Silvestre, G. Simatovic, R. Singaraju, R. Singh, S. Singha, V. Singhal, B. C. Sinha, T. Sinha, B. Sitar, M. Sitta, T. B. Skaali, K. Skjerdal, R. Smakal, N. Smirnov, R. J. M. Snellings, C. Søgaard, R. Soltz, M. Song, J. Song, C. Soos, F. Soramel, M. Spacek, I. Sputowska, M. Spyropoulou-Stassinaki, B. K. Srivastava, J. Stachel, I. Stan, G. Stefanek, M. Steinpreis, E. Stenlund, G. Steyn, J. H. Stiller, D. Stocco, M. Stolpovskiy, P. Strmen, A. A. P. Suaide, M. A. Subieta Vásquez, T. Sugitate, C. Suire, M. Suleymanov, R. Sultanov, M. Šumbera, T. Susa, T. J. M. Symons, A. Szanto de Toledo, I. Szarka, A. Szczepankiewicz, M. Szymański, J. Takahashi, M. A. Tangaro, J. D. Tapia Takaki, A. Tarantola Peloni, A. Tarazona Martinez, A. Tauro, G. Tejeda Muñoz, A. Telesca, C. Terrevoli, A. Ter Minasyan, J. Thäder, D. Thomas, R. Tieulent, A. R. Timmins, A. Toia, H. Torii, V. Trubnikov, W. H. Trzaska, T. Tsuji, A. Tumkin, R. Turrisi, T. S. Tveter, J. Ulery, K. Ullaland, J. Ulrich, A. Uras, G. M. Urciuoli, G. L. Usai, M. Vajzer, M. Vala, L. Valencia Palomo, P. Vande Vyvre, L. Vannucci, J. W. Van Hoorne, M. van Leeuwen, A. Vargas, R. Varma, M. Vasileiou, A. Vasiliev, V. Vechernin, M. Veldhoen, M. Venaruzzo, E. Vercellin, S. Vergara, R. Vernet, M. Verweij, L. Vickovic, G. Viesti, J. Viinikainen, Z. Vilakazi, O. Villalobos Baillie, A. Vinogradov, L. Vinogradov, Y. Vinogradov, T. Virgili, Y. P. Viyogi, A. Vodopyanov, M. A. Völkl, S. Voloshin, K. Voloshin, G. Volpe, B. von Haller, I. Vorobyev, D. Vranic, J. Vrláková, B. Vulpescu, A. Vyushin, B. Wagner, V. Wagner, J. Wagner, Y. Wang, Y. Wang, M. Wang, D. Watanabe, K. Watanabe, M. Weber, J. P. Wessels, U. Westerhoff, J. Wiechula, J. Wikne, M. Wilde, G. Wilk, J. Wilkinson, M. C. S. Williams, B. Windelband, M. Winn, C. Xiang, C. G. Yaldo, Y. Yamaguchi, H. Yang, P. Yang, S. Yang, S. Yano, S. Yasnopolskiy, J. Yi, Z. Yin, I.-K. Yoo, I. Yushmanov, V. Zaccolo, C. Zach, C. Zampolli, S. Zaporozhets, A. Zarochentsev, P. Závada, N. Zaviyalov, H. Zbroszczyk, P. Zelnicek, I. S. Zgura, M. Zhalov, F. Zhang, Y. Zhang, H. Zhang, X. Zhang, D. Zhou, Y. Zhou, F. Zhou, X. Zhu, J. Zhu, J. Zhu, H. Zhu, A. Zichichi, M. B. Zimmermann, A. Zimmermann, G. Zinovjev, Y. Zoccarato, M. Zynovyev, M. Zyzak

**Affiliations:** 1 CERN, 1211 Geneva 23, Switzerland; 2 A.I. Alikhanyan National Science Laboratory (Yerevan Physics Institute) Foundation, Yerevan, Armenia; 3 Benemérita Universidad Autónoma de Puebla, Puebla, Mexico; 4 Bogolyubov Institute for Theoretical Physics, Kiev, Ukraine; 5 Budker Institute for Nuclear Physics, Novosibirsk, Russia; 6 California Polytechnic State University, San Luis Obispo, California United States; 7 Central China Normal University, Wuhan, China; 8 Centre de Calcul de l’IN2P3, Villeurbanne, France; 9 Centro de Aplicaciones Tecnológicas y Desarrollo Nuclear (CEADEN), Havana, Cuba; 10 Centro de Investigaciones Energéticas Medioambientales y Tecnológicas (CIEMAT), Madrid, Spain; 11 Centro de Investigación y de Estudios Avanzados (CINVESTAV), Mexico City and Mérida, Mexico; 12 Centro Fermi - Museo Storico della Fisica e Centro Studi e Ricerche “Enrico Fermi”, Rome, Italy; 13 Chicago State University, Chicago, United States; 14 Commissariat à l’Energie Atomique, IRFU, Saclay, France; 15 COMSATS Institute of Information Technology (CIIT), Islamabad, Pakistan; 16 Departamento de Física de Partículas and IGFAE, Universidad de Santiago de Compostela, Santiago de Compostela, Spain; 17 Department of Physics, Aligarh Muslim University, Aligarh, India; 18 Department of Physics and Technology, University of Bergen, Bergen, Norway; 19 Department of Physics, Ohio State University, Columbus, Ohio United States; 20 Department of Physics, Sejong University, Seoul, South Korea; 21 Department of Physics, University of Oslo, Oslo, Norway; 22 Dipartimento di Fisica dell’Università and Sezione INFN, Cagliari, Italy; 23 Dipartimento di Fisica dell’Università and Sezione INFN, Trieste, Italy; 24 Dipartimento di Fisica dell’Università and Sezione INFN, Turin, Italy; 25 Dipartimento di Fisica dell’Università ‘La Sapienza’ and Sezione INFN, Rome, Italy; 26 Dipartimento di Fisica e Astronomia dell’Università and Sezione INFN, Bologna, Italy; 27 Dipartimento di Fisica e Astronomia dell’Università and Sezione INFN, Catania, Italy; 28 Dipartimento di Fisica e Astronomia dell’Università and Sezione INFN, Padova, Italy; 29 Dipartimento di Fisica ‘E.R. Caianiello’ dell’Università and Gruppo Collegato INFN, Salerno, Italy; 30 Dipartimento di Scienze e Innovazione Tecnologica dell’Università del Piemonte Orientale and Gruppo Collegato INFN, Alessandria, Italy; 31 Dipartimento Interateneo di Fisica ‘M. Merlin’ and Sezione INFN, Bari, Italy; 32 Division of Experimental High Energy Physics, University of Lund, Lund, Sweden; 33 Eberhard Karls Universität Tübingen, Tübingen, Germany; 34 European Organization for Nuclear Research (CERN), Geneva, Switzerland; 35 Faculty of Engineering, Bergen University College, Bergen, Norway; 36 Faculty of Mathematics, Physics and Informatics, Comenius University, Bratislava, Slovakia; 37 Faculty of Nuclear Sciences and Physical Engineering, Czech Technical University in Prague, Prague, Czech Republic; 38 Faculty of Science, P.J. Šafárik University, Košice, Slovakia; 39 Frankfurt Institute for Advanced Studies, Johann Wolfgang Goethe-Universität Frankfurt, Frankfurt, Germany; 40 Gangneung-Wonju National University, Gangneung, South Korea; 41 Helsinki Institute of Physics (HIP), Helsinki, Finland; 42 Hiroshima University, Hiroshima, Japan; 43 Indian Institute of Technology Bombay (IIT), Mumbai, India; 44 Indian Institute of Technology Indore (IITI), Indore, India; 45 Institut de Physique Nucléaire d’Orsay (IPNO), Université Paris-Sud, CNRS-IN2P3, Orsay, France; 46 Institut für Informatik, Johann Wolfgang Goethe-Universität Frankfurt, Frankfurt, Germany; 47 Institut für Kernphysik, Johann Wolfgang Goethe-Universität Frankfurt, Frankfurt, Germany; 48 Institut für Kernphysik, Technische Universität Darmstadt, Darmstadt, Germany; 49 Institut für Kernphysik, Westfälische Wilhelms-Universität Münster, Münster, Germany; 50 Institut Pluridisciplinaire Hubert Curien (IPHC), Université de Strasbourg, CNRS-IN2P3, Strasbourg, France; 51 Institute for High Energy Physics, Protvino, Russia; 52 Institute for Nuclear Research, Academy of Sciences, Moscow, Russia; 53 Institute for Subatomic Physics of Utrecht University, Utrecht, Netherlands; 54 Institute for Theoretical and Experimental Physics, Moscow, Russia; 55 Institute of Experimental Physics, Slovak Academy of Sciences, Košice, Slovakia; 56 Institute of Physics, Academy of Sciences of the Czech Republic, Prague, Czech Republic; 57 Institute of Physics, Bhubaneswar, India; 58 Institute of Space Science (ISS), Bucharest, Romania; 59 Instituto de Ciencias Nucleares, Universidad Nacional Autónoma de México, Mexico City, Mexico; 60 Instituto de Física, Universidad Nacional Autónoma de México, Mexico City, Mexico; 61 iThemba LABS, National Research Foundation, Somerset West, South Africa; 62 Joint Institute for Nuclear Research (JINR), Dubna, Russia; 63 Korea Institute of Science and Technology Information, Daejeon, South Korea; 64 KTO Karatay University, Konya, Turkey; 65 Laboratoire de Physique Corpusculaire (LPC), Clermont Université, Université Blaise Pascal, CNRS–IN2P3, Clermont-Ferrand, France; 66 Laboratoire de Physique Subatomique et de Cosmologie (LPSC), Université Joseph Fourier, Institut Polytechnique de Grenoble, CNRS-IN2P3, Grenoble, France; 67 Laboratori Nazionali di Frascati, INFN, Frascati, Italy; 68 Laboratori Nazionali di Legnaro, INFN, Legnaro, Italy; 69 Lawrence Berkeley National Laboratory, Berkeley, California United States; 70 Lawrence Livermore National Laboratory, Livermore, California United States; 71 Moscow Engineering Physics Institute, Moscow, Russia; 72 National Centre for Nuclear Studies, Warsaw, Poland; 73 National Institute for Physics and Nuclear Engineering, Bucharest, Romania; 74 National Institute of Science Education and Research, Bhubaneswar, India; 75 Niels Bohr Institute, University of Copenhagen, Copenhagen, Denmark; 76 Nikhef, National Institute for Subatomic Physics, Amsterdam, Netherlands; 77 Nuclear Physics Group, STFC Daresbury Laboratory, Daresbury, United Kingdom; 78 Nuclear Physics Institute, Academy of Sciences of the Czech Republic, Řež u Prahy, Czech Republic; 79 Oak Ridge National Laboratory, Oak Ridge, Tennessee United States; 80 Petersburg Nuclear Physics Institute, Gatchina, Russia; 81 Physics Department, Creighton University, Omaha, Nebraska United States; 82 Physics Department, Panjab University, Chandigarh, India; 83 Physics Department, University of Athens, Athens, Greece; 84 Physics Department, University of Cape Town, Cape Town, South Africa; 85 Physics Department, University of Jammu, Jammu, India; 86 Physics Department, University of Rajasthan, Jaipur, India; 87 Physikalisches Institut, Ruprecht-Karls-Universität Heidelberg, Heidelberg, Germany; 88 Politecnico di Torino, Turin, Italy; 89 Purdue University, West Lafayette, Indiana United States; 90 Pusan National University, Pusan, South Korea; 91 Research Division and ExtreMe Matter Institute EMMI, GSI Helmholtzzentrum für Schwerionenforschung, Darmstadt, Germany; 92 Rudjer Bošković Institute, Zagreb, Croatia; 93 Russian Federal Nuclear Center (VNIIEF), Sarov, Russia; 94 Russian Research Centre Kurchatov Institute, Moscow, Russia; 95 Saha Institute of Nuclear Physics, Kolkata, India; 96 School of Physics and Astronomy, University of Birmingham, Birmingham, United Kingdom; 97 Sección Física, Departamento de Ciencias, Pontificia Universidad Católica del Perú, Lima, Peru; 98 Sezione INFN, Bari, Italy; 99 Sezione INFN, Bologna, Italy; 100 Sezione INFN, Cagliari, Italy; 101 Sezione INFN, Catania, Italy; 102 Sezione INFN, Padova, Italy; 103 Sezione INFN, Rome, Italy; 104 Sezione INFN, Trieste, Italy; 105 Sezione INFN, Turin, Italy; 106 SUBATECH, Ecole des Mines de Nantes, Université de Nantes, CNRS-IN2P3, Nantes, France; 107 Technical University of Split FESB, Split, Croatia; 108 The Henryk Niewodniczanski Institute of Nuclear Physics, Polish Academy of Sciences, Cracow, Poland; 109 Physics Department, The University of Texas at Austin, Austin, TX United States; 110 Universidad Autónoma de Sinaloa, Culiacán, Mexico; 111 Universidade de São Paulo (USP), São Paulo, Brazil; 112 Universidade Estadual de Campinas (UNICAMP), Campinas, Brazil; 113 University of Houston, Houston, Texas United States; 114 University of Jyväskylä, Jyväskylä, Finland; 115 University of Tennessee, Knoxville, Tennessee United States; 116 University of Tokyo, Tokyo, Japan; 117 University of Tsukuba, Tsukuba, Japan; 118 Université de Lyon, Université Lyon 1, IPN-Lyon, CNRS/IN2P3, Villeurbanne, France; 119 V. Fock Institute for Physics, St. Petersburg State University, St. Petersburg, Russia; 120 Variable Energy Cyclotron Centre, Kolkata, India; 121 Vestfold University College, Tonsberg, Norway; 122 Warsaw University of Technology, Warsaw, Poland; 123 Wayne State University, Detroit, Michigan United States; 124 Wigner Research Centre for Physics, Hungarian Academy of Sciences, Budapest, Hungary; 125 Yale University, New Haven, Connecticut United States; 126 Yonsei University, Seoul, South Korea; 127 Zentrum für Technologietransfer und Telekommunikation (ZTT), Fachhochschule Worms, Worms, Germany

## Abstract

Differential cross sections of charged particles in inelastic pp collisions as a function of *p*_T_ have been measured at $\sqrt{s} = 0.9,\ 2.76\ \text{and}\ 7\ \text{TeV}$ at the LHC. The *p*_T_ spectra are compared to NLO-pQCD calculations. Though the differential cross section for an individual $\sqrt{s}$ cannot be described by NLO-pQCD, the relative increase of cross section with $\sqrt{s}$ is in agreement with NLO-pQCD. Based on these measurements and observations, procedures are discussed to construct pp reference spectra at $\sqrt{s} = 2.76\ \text{and}\ 5.02~\text{TeV}$ up to *p*_T_=50 GeV/*c* as required for the calculation of the nuclear modification factor in nucleus–nucleus and proton–nucleus collisions.

## Introduction

The measurement of charged particle production in proton–proton collisions at high energy gives insight into the dynamics of soft and hard interactions. Hard parton–parton scattering processes with large momentum transfer are quantitatively described by perturbative Quantum Chromodynamics (pQCD). Measurements at high transverse momenta (*p*_T_) at LHC-energies can help to constrain parton distribution and fragmentation functions in current next-to-Leading-Order (NLO) pQCD calculations [1] of charged particle production. As data at various $\sqrt{s}$ become available at the LHC, a systematic comparison with current NLO-pQCD calculations over a large span of $\sqrt{s}$ is now possible. However, most particles are produced at low momentum, where particle production is dominated by soft interactions and only phenomenological approaches can be applied (e.g. PYTHIA [2–4], PHOJET [5]) to describe the data. A systematic comparison to data at different values of $\sqrt{s}$ is an essential ingredient to tune these Monte Carlo event generators.

Furthermore, the measurement of charged particle transverse momentum spectra in pp collisions serves as a crucial reference for particle spectra in Pb–Pb collisions. To quantify final state effects due to the creation of a hot and dense deconfined matter, commonly referred to as the Quark–Gluon Plasma (QGP), *p*_T_ spectra in the two collision systems are compared. The observed suppression [6] in central Pb–Pb collisions at LHC-energies at high *p*_T_ relative to an independent superposition of pp collisions is generally attributed to energy loss of the partons as they propagate through the hot and dense QCD medium. To enable this comparison a pp reference *p*_T_ spectrum at the same $\sqrt{s}$ with the same *p*_T_ coverage has to be provided. Similarly, a pp reference spectrum is also needed for p–Pb collisions to investigate possible initial-state effects in the collision.

In this paper we present a measurement of primary charged particle transverse momentum spectra in pp collisions at $\sqrt{s} = 0.9,\ 2.76 \ \mbox{and}\ 7\ \text{TeV}$. Primary charged particles are considered here as all charged particles produced in the collision and their decay products, except for particles from weak decays of strange hadrons. The measurement is performed in the pseudorapidity range |*η*|<0.8 for particles with *p*_T_>0.15 GeV/*c*. Reference spectra for comparison with Pb–Pb spectra at $\sqrt {s_{\mathrm{NN}}} = 2.76\ \mbox{TeV}$ and p–Pb spectra at $\sqrt{s_{\mathrm {NN}}} = 5.02\ \mbox{TeV}$ in the corresponding *p*_T_ range up to *p*_T_=50 GeV/*c* are constructed.

## Experiment and data analysis

The data were collected by the ALICE apparatus [7] at the CERN-LHC in 2009–2011. The analysis is based on tracking information from the Inner Tracking System (ITS) and the Time Projection Chamber (TPC), both located in the central barrel of the experiment. The minimum-bias interaction trigger was derived using signals from the forward scintillators (VZERO), and the two innermost layers of the ITS, the Silicon Pixel Detector (SPD). Details of the experimental setup used in this analysis are discussed in [8].

The events are selected based on the minimum-bias trigger MB_OR_ requiring at least one hit in the SPD or VZERO detectors, which are required to be in coincidence with two beam bunches crossing in the ALICE interaction region. In addition, an offline event selection is applied to reject beam induced (beam-gas, beam-halo) background. The VZERO counters are used to remove these beam-gas or beam-halo events by requiring their timing signals to be in coincidence with particles produced in the collision. The background events are also removed by exploiting the correlation between the number of the SPD hits and the number of the SPD tracklets (short track segments reconstructed in the SPD and pointing to the interaction vertex). The beam-gas or beam-halo events typically have a large number of hits in the SPD compared to the number of reconstructed tracklets; this is used to reject background events. In total 6.8 M, 65 M and 150 M pp events at $\sqrt{s}=0.9,\ 2.76\ \mbox{and}\ 7\ \text{TeV}$ fulfill the $\rm {MB_{{OR}}}$ trigger and offline selection criteria. The typical luminosity for these data taking was about 10^29^ s^−1^ cm^−2^. The average number of interactions per bunch crossing varied from 0.05 to 0.1.

In this analysis the focus is on inelastic (INEL) pp events originating from single-diffractive, double-diffractive and non-diffractive processes. The INEL events are selected with an efficiency $\varepsilon _{\mathrm{MB}_{\mathrm{OR}}}$ of $91^{+3.2}_{-1.0}~\%$, $88.1^{+5.9}_{-3.5}~\%$ and $85.2^{+6.2}_{-3.0}~\%$ for the three energies. The trigger efficiencies are determined [9] based on detector simulations with PYTHIA6 [2–4] and PHOJET [5] event generators.

The primary event vertex is determined based on ITS and TPC information. If no vertex is found using tracks in the ITS and the TPC, it is reconstructed from tracklets in the SPD only. Tracks or tracklets are extrapolated to the experimental collision region utilizing the averaged measured beam intersection profile in the *x*–*y* plane perpendicular to the beam axis.

An event is accepted if the *z*-coordinate of the vertex is within ±10 cm of the center of the interaction region along the beam direction. This corresponds to about 1.6 standard deviations from the mean of the reconstructed event vertex distribution for all three energies. In this range, the vertex reconstruction efficiency is independent of *z*. The event vertex reconstruction is fully efficient for events with at least one track in the pseudorapidity range |*η*|<1.4 for all three energies.

Only tracks within a pseudorapidity range of |*η*|<0.8 and transverse momenta *p*_T_>0.15 GeV/*c* are selected. A set of standard cuts based on the number of space points and the quality of the track fit in ITS and TPC is applied to the reconstructed tracks [10].

Efficiency and purity of the primary charged particle selection are estimated using simulations with PYTHIA6 [2–4] and GEANT3 [11] for particle transport and detector response. The overall *p*_T_-dependent efficiency (tracking efficiency × acceptance) is 40–73 %, 36–68 % and 40–73 % at $\sqrt{s}=0.9,\ 2.76\ \mbox{and}\ 7\ \text{TeV}$. At $\sqrt{s}=2.76~\text{TeV}$ the overall efficiency is lower than at $\sqrt {s}=0.9\ \text{and}\ 7\ \text{TeV}$ due to the smaller number of operational channels in the SPD. Contamination of secondary tracks which passed all selection criteria amounts to 7 % at *p*_T_=0.15 GeV/*c* and decreases to ∼0.6 % for *p*_T_>4 GeV/*c*. In addition, the contribution from secondary tracks originating from weak decays of strange hadrons was scaled up by a factor of 1–1.5 (*p*_T_-dependent) to match the contribution in data. The secondary tracks were subtracted bin-by-bin from the *p*_T_ spectra.

The *p*_T_ resolution is estimated from the space point residuals of the track fit. It is verified by the width of the invariant mass peaks of *Λ*, $\overline{\varLambda}$ and $\mathrm{K}^{0}_{\mathrm{{s}}}$, reconstructed from their decays into two charged particles. The relative *p*_T_ resolution is 3.5 %, 5.5 % and 9 % at the highest *p*_T_ of 20, 32 and 50 GeV/*c* at $\sqrt{s}=0.9,\ 2.76\ \text{and}\ 7~\text{TeV}$, respectively. From invariant mass distributions *M*_inv_(*p*_T_) of *Λ* and $\mathrm{K}^{0}_{\mathrm{{s}}}$, the relative uncertainty on the *p*_T_ resolution is estimated to be ≈20 % for all three energies. To account for the finite *p*_T_ resolution of tracks, correction factors to the spectrum for *p*_T_>10 GeV/*c* are derived using an unfolding procedure. The determination of the correction factors is based on measured tracks without involving simulation. The choice of the unfolding procedure is based on the observation that *p*_T_ smearing has a small influence on the measured spectrum. As input to the procedure a power-law parametrization of the measured *p*_T_ spectrum for *p*_T_>10 GeV/*c* is used. This parametrization is folded with the *p*_T_ resolution obtained for a given *p*_T_ from the measured track covariance matrix. The *p*_T_ dependent correction factors are extracted from the ratio of the input to the folded parametrization and are applied (bin-by-bin) to the measured *p*_T_ spectrum. It was checked that the derived correction factors are the same when replacing the measured with the corrected *p*_T_ distribution in the unfolding procedure. The correction factors depend on $\sqrt{s}$ due to the change of the spectral shape and reach 2 %, 4 % and 6.5 % at $\sqrt{s}=0.9,~2.76~\text{and}~7~\text{TeV}$ for the highest *p*_T_. The systematic uncertainty of the momentum scale is |Δ(*p*_T_)/*p*_T_|<0.01 at *p*_T_=50 GeV/*c*, as determined from the mass difference between *Λ* and $\overline{\varLambda}$ and the ratio of positively to negatively charged tracks, assuming charge symmetry at high *p*_T_.

**Fig. 1:**
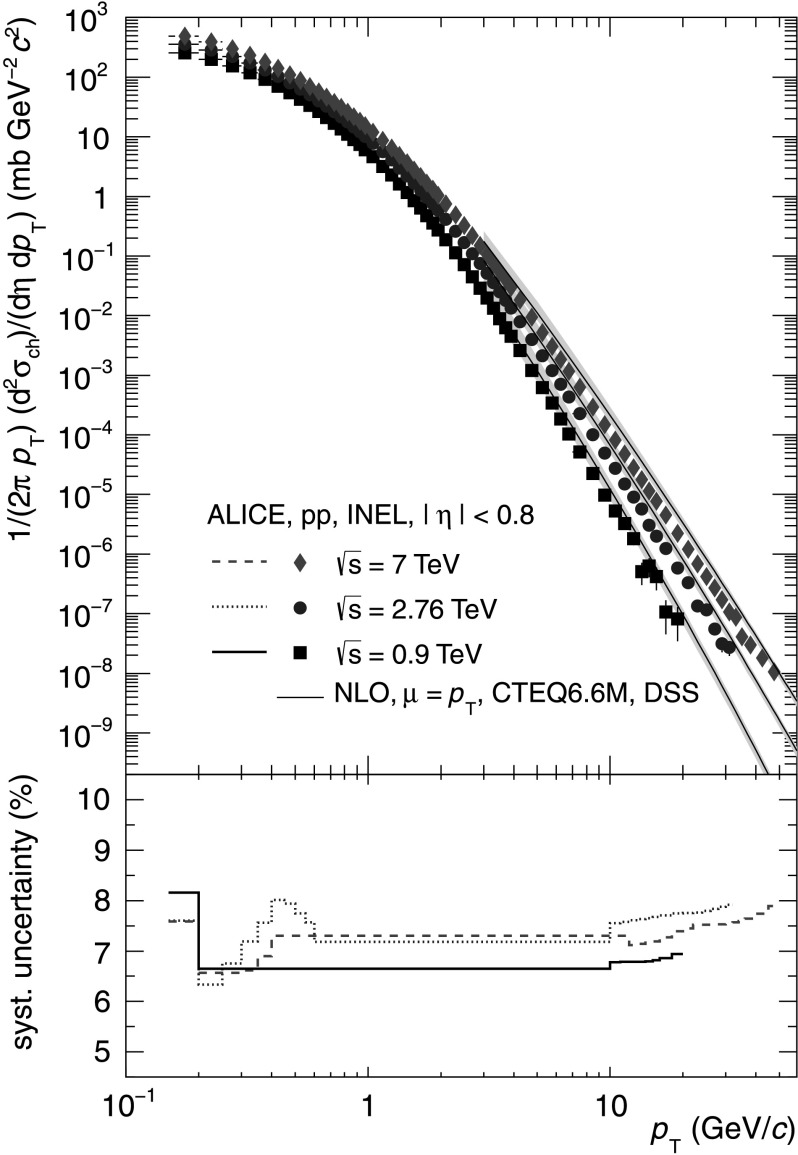
*Top*: Differential cross section of charged particles in INEL pp collisions at $\sqrt{s} = 0.9,\ 2.76\ \text{and}\ 7~\text{TeV}$ as a function of *p*_T_ compared to a NLO-pQCD calculation [1] at the same energy. Only statistical uncertainties are shown. *Bottom*: Systematic uncertainties as a function of *p*_T_ for all three energies. The uncertainty on the normalization (compare Table 1) of the spectra is not included (Color figure online)

A summary of the systematic uncertainties is given in Table 1. The systematic uncertainties on the event selection are determined by changing the lower and upper limits on the *z*-coordinate of the vertex. Track selection criteria [10] are varied to determine the corresponding systematic uncertainties resulting in a maximal contribution of 4.3–5.5 % for *p*_T_<0.6 GeV/*c*. The systematic uncertainties on the tracking efficiency are estimated from the difference between data and simulation in the TPC-ITS track matching efficiency. The systematic uncertainties related to the *p*_T_ resolution correction are derived from the unfolding procedure including a relative uncertainty on the *p*_T_ resolution, and reach maximum values at the highest *p*_T_ covered. The systematic uncertainties on the material budget (∼11.5 % *X*_0_ [12], where *X*_0_ is the radiation length) are estimated by changing the material density (conservatively) by ±10 % in the simulation, contributing mostly at *p*_T_<0.2 GeV/*c*. To assess the systematic uncertainties on the tracking efficiency related to the primary particle composition the relative abundance of *π*, K, p was varied by 30 % in the simulation; they contribute mostly at *p*_T_<0.5 GeV/*c*. The Monte Carlo (MC) event generator dependence was studied using PHOJET as a comparison, with the largest contribution at *p*_T_<0.2 GeV/*c*. The yield of secondary particles from decays of strange hadrons has been varied by 30 % to determine the corresponding uncertainty of maximum 0.3 % at *p*_T_≈1 GeV/*c*. The total *p*_T_ dependent systematic uncertainties for the three energies amount to 6.7–8.2 %, 6.4–8.0 % and 6.6–7.9 % and are shown in the bottom panel of Fig. 1. They are dominated by the systematic uncertainties on the tracking efficiency. There are also comparable contributions related to the track selection (*p*_T_<0.6 GeV/*c*) and *p*_T_ resolution correction at the highest *p*_T_ covered. The systematic uncertainties on the normalization are related to the minimum bias nucleon–nucleon cross section ($\sigma^{\mathrm {NN}}_{\mathrm{MB}}$) determination [9] and amount to +5.1/−4.0 %, ±1.9 % and ±3.6 % for pp at $\sqrt{s}=0.9~\mbox{TeV},\ 2.76~\text{TeV}\ \mbox{and}\ 7~\text{TeV}$, respectively.

**Table 1:** Contribution to the systematic uncertainties on the *p*_T_ spectra

$\sqrt{s}$	0.9 TeV	2.76 TeV	7 TeV
Event vertex selection	1.2 %	2.3 %	0.5 %
Track selection	2.5–5.5 %	2.3–5.1 %	1.9–4.3 %
Tracking efficiency	5 %	5 %	5 %
*p*_T_ resolution correction	<1.7 %	<1.9 %	<2.6 %
Material budget	0.2–1.5 %	0.2–1.5 %	0.2–1.5 %
Particle composition	1–2 %	1–2 %	1–2 %
MC event generator	2.5 %	2–3 %	2–3.5 %
Secondary strange particles	<0.3 %	<0.3 %	<0.3 %
Total *p*_T_ dependent	6.7–8.2 %	6.4–8.0 %	6.6–7.9 %
Normalization uncertainty	+5.1/−4.0 %	±1.9 %	±3.6 %

The differential cross section d^2^ *σ*_ch_/d*η* d*p*_T_ is calculated as $\mathrm{d}^{2}\sigma_{\mathrm{ch}} / {\mathrm{d}}\eta\,\mathrm {d}p_{\mathrm{T}} = \sigma_{\mathrm{MB}_{\mathrm {OR}}}^{\mathrm{NN}} \times\mathrm{d}^{2} {N}^{\mathrm {MB}_{\mathrm{OR}}}_{\mathrm{{ch}}} / {\mathrm{{d}}} \eta \,{\mathrm{{d}}}p_{\mathrm{T}}$ with $\mathrm{{d}}^{2} {{N}}^{\mathrm{{MB_{\mathrm{{OR}}}}}}_{\mathrm {{ch}}} / {\mathrm{{d}}} \eta\,{\mathrm{{d}}}p_{\mathrm{T}}$ being the per event differential yield of charged particles in minimum bias collisions. $\sigma_{\mathrm{MB}_{\mathrm{OR}}}^{\mathrm{{NN}}}$ is determined based on van-der-Meer scans [9] as $\sigma_{\mathrm{MB}_{\mathrm{OR}}}^{\mathrm{NN}} = 55.4 \pm1.0$ (62.2±2.2) mb at $\sqrt{s}=2.76~(7)~\mbox{TeV}$. At $\sqrt{s}=0.9~\mbox{TeV}$ van-der-Meer scans were not performed and $\sigma_{\mathrm{MB}_{\mathrm{OR}}}^{\mathrm {{NN}}}=47.8^{+2.5}_{-3.0}~\mbox{mb}$ is obtained based on detector simulations using the INEL cross section $\sigma^{\mathrm{{NN}}}_{\mathrm{{INEL}}}=52.5^{+2}_{-3.3}~\mbox{mb}$ [9]. $\sigma^{\mathrm{{NN}}}_{\mathrm{{INEL}}}$ includes the UA5 measurement [13] and re-analysis of the extrapolation to low diffractive masses [14].

## Results

The differential cross section in INEL pp collisions as a function of *p*_T_ is shown in Fig. 1 for all three measured collision energies. At high *p*_T_ a clear evolution of the slope from $\sqrt {s}= 0.9\ \mbox{to}\ 7~\text{TeV}$ can be observed. A NLO-pQCD calculation [1] for *p*_T_>3 GeV/*c* is compared to the spectra. The calculation shows a similar evolution of the high-*p*_T_ dependence with $\sqrt{s}$ but overpredicts the data by a factor two [12, 15]. The low systematic uncertainties demonstrate the accuracy of the measurements for all energies over the full *p*_T_ range.

**Fig. 2:**
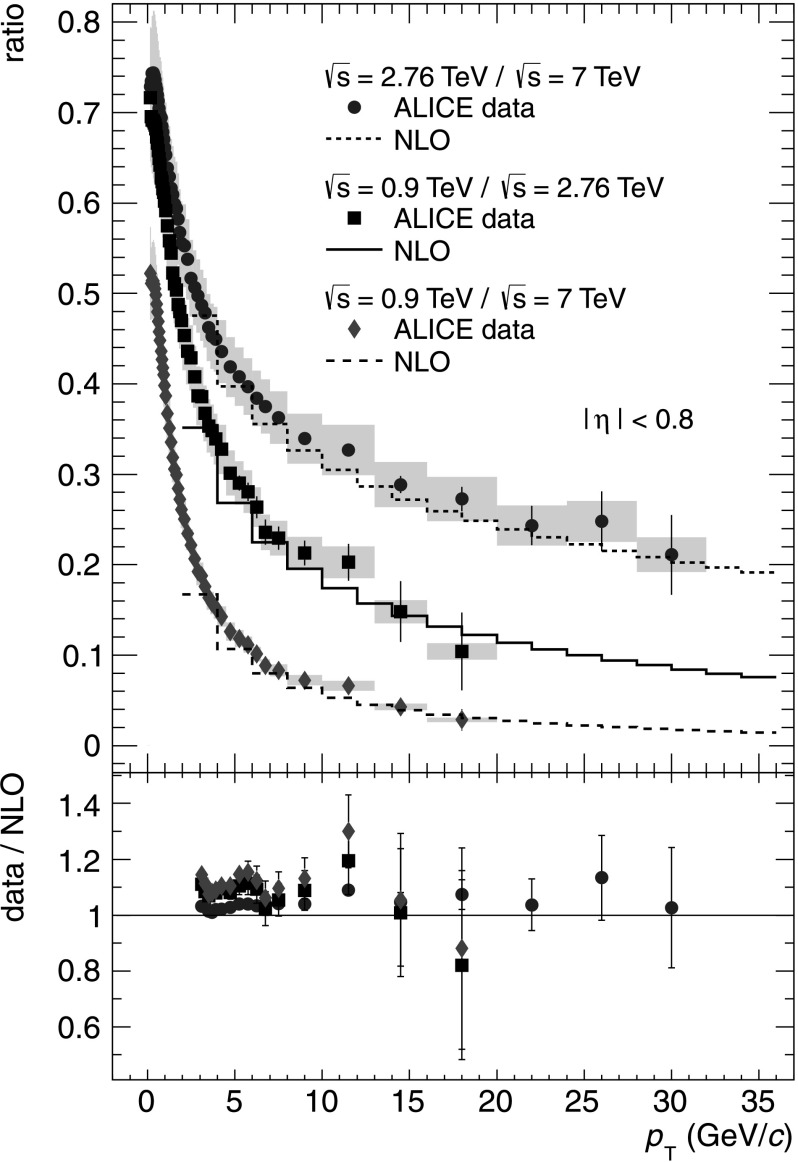
*Top*: Ratio of differential cross sections of charged particles in INEL pp collisions at different collision energies as a function of *p*_T_. *Gray boxes* denote *p*_T_ dependent systematic uncertainties. Normalization uncertainties are not shown (see *text* for details). The *histograms* show the same ratio determined from NLO calculations. *Bottom*: Ratio of data and NLO calculations derived from *upper panel*. A variation of the renormalization and factorization scale of the NLO calculation gives a systematic uncertainty on the double ratio of 0.5–23.6 % for 0.9 TeV/2.76 TeV, 1.0–37.8 % for 0.9 TeV/7 TeV and 2.4–12.3 % for 2.76 TeV/7 TeV (Color figure online)

Though the *p*_T_ dependence of the cross section for a single $\sqrt{s}$ is not well described by NLO-pQCD, the relative dependence on *p*_T_ of cross sections of two collision energies is described much better. Figure 2 shows the ratio between the differential cross section in INEL pp collisions at $\sqrt{s} = 2.76\ \mbox{to}\ 7~\mbox{TeV},\ 0.9\ \mbox{to}\ 2.76~\mbox{TeV}\ \mbox{and}\ 0.9\ \mbox{to}\ 7~\mbox{TeV}$ as a function of *p*_T_ in comparison to the same ratio calculated with NLO-pQCD. The total *p*_T_ dependent systematic uncertainties on the ratios are evaluated taking into account correlated contributions, and amount to 8.1–9.8 %, 7.8–9.8 % and 7.9–9.9 % for 0.9 TeV/2.76 TeV, 0.9 TeV/7 TeV and 2.76 TeV/7 TeV. The corresponding normalization uncertainties amount to +5.4 %/−4.4 %, +6.2 %/−5.4 % and ±4.1 %, and are calculated assuming that the normalization uncertainties on the *p*_T_ spectra (Table 1) are uncorrelated. In all three ratios good agreement between data and NLO-pQCD calculations is found, which can be seen in the double ratio of data and NLO-pQCD for the three energy ratios in the lower panel of Fig. 2.

## Construction of a pp reference for $\sqrt{s} = 2.76\ \mbox{TeV}$

For the determination of the nuclear modification factor

1 $$ R_{\mathrm{AA}} (p_{\mathrm{T}}) = \frac{ \mathrm{d}^2 {N} _{\mathrm{ch}}^{\mathrm{AA}}/ {\mathrm{d}} \eta\,{\mathrm {d}}p_{\mathrm{T}}}{ \langle T_{\mathrm{AA}} \rangle\, \mathrm{d}^2 \sigma_{\mathrm {ch}}^{\mathrm{pp}} / {\mathrm{d}} \eta\,{\mathrm{d}}p_{\mathrm{T}}} $$

in heavy-ion collisions a well described pp reference $\mathrm{d}^{2} \sigma_{\mathrm {ch}}^{\mathrm{pp}} / {\mathrm{d}} \eta\,{\mathrm{d}}p_{\mathrm{T}}$ at the same center-of-mass energy up to high *p*_T_ is essential. ${N}_{\mathrm{ch}}^{\mathrm{AA}}$ describes the charged particle yield per event in nucleus–nucleus collisions and 〈*T*_AA_〉 is the average nuclear overlap function [6, 10]. The statistics in the measurement of $\mathrm{d}^{2} \sigma_{\mathrm {ch}}^{\mathrm{pp}} / {\mathrm{d}} \eta\,{\mathrm{d}}p_{\mathrm{T}}$ for $\sqrt{s} = 2.76\ \mbox{TeV}$ reported in this paper allows *p*_T_=32 GeV/*c* to be reached. In order to extrapolate to higher *p*_T_, the measured cross section needs to be parametrized.

As can be seen in Fig. 1 for *p*_T_>10 GeV/*c* the pp spectrum at $\sqrt{s} = 2.76\ \mbox{TeV}$ shows a clear power-law dependence on *p*_T_. To constrain the parametrization better by including data points at lower *p*_T_, $\mathrm{d}^{2} \sigma_{\mathrm{ch}}^{\mathrm{pp}} / {\mathrm{d}} \eta\,{\mathrm{d}}p_{\mathrm{T}}$ has been parametrized by a so-called modified Hagedorn function [16]

2 $$ \frac{1}{2\pi p_{\mathrm{T}}}\frac{\mathrm{d}^2 \sigma_{\mathrm {ch}}^{\mathrm{pp}}}{\mathrm{d} \eta\,{\mathrm{d}}p_{\mathrm{T}}} = A \frac{p_{\mathrm{T}}}{m_{\mathrm{T}}} \biggl(1+ \frac{p_{\mathrm {T}}}{p_{\mathrm{T,0}}} \biggr)^{-n} $$

where *m*_T_ denotes the transverse mass $m_{\mathrm{T}} = \sqrt{m_{0}^{2} + p_{\mathrm{T}}^{2}}$, with *m*_0_=140 MeV/*c* assumed for all tracks. For small *p*_T_, the term $(1+ \frac {p_{\mathrm{T}}}{p_{\mathrm{T,0}}} )^{-n}$ behaves like an exponential function with an inverse slope parameter of $p_{\mathrm{T,0}}/n$ while for large *p*_T_ the Hagedorn function behaves like a power-law function.

To determine the extrapolation to high *p*_T_, $\mathrm {d}^{2} \sigma_{\mathrm{ch}}^{\mathrm{pp}} / {\mathrm{d}} \eta \,{\mathrm{d}}p_{\mathrm{T}}$ is parametrized for *p*_T_>5 GeV/*c*. For 5 GeV/*c*<*p*_T_<10 GeV/*c* the exponential part of the Hagedorn function acts as a correction term to the power-law part in the function.

**Fig. 3:**
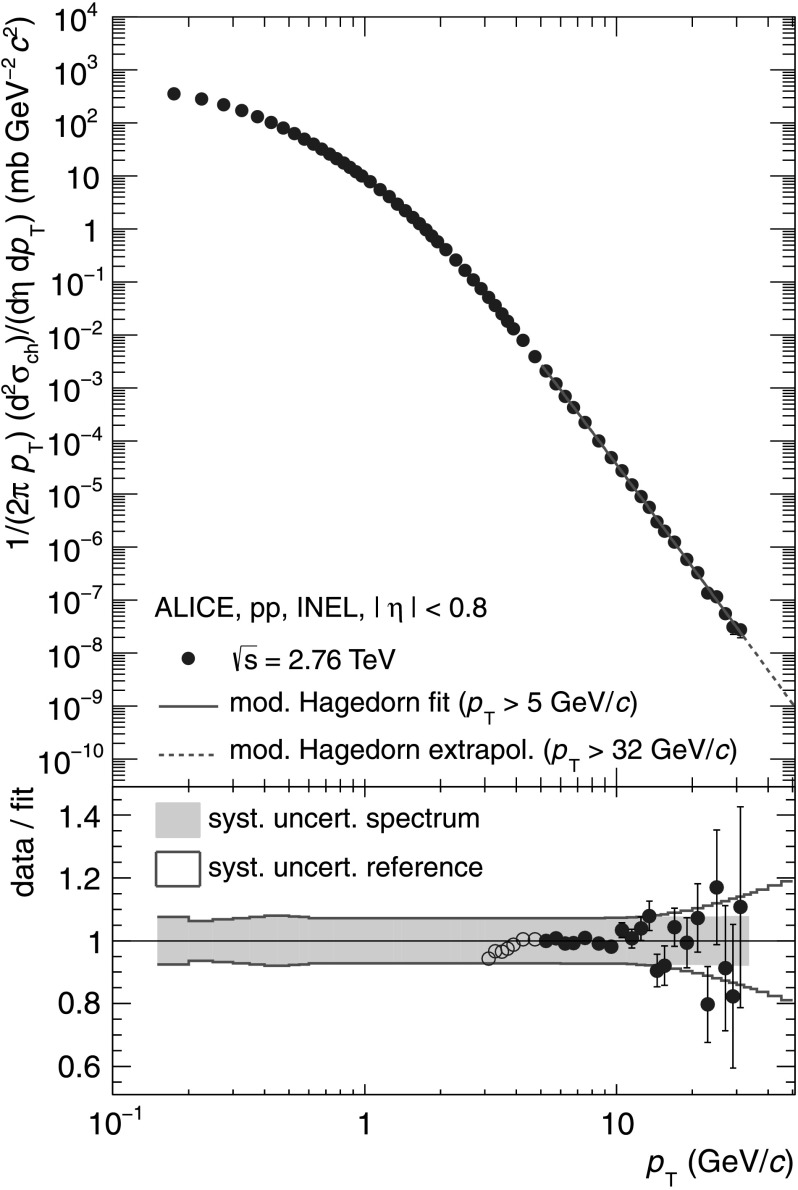
*Top*: Differential cross section of charged particles in INEL pp collisions at $\sqrt{s} = 2.76\ \mbox{TeV}$ as a function of *p*_T_ together with the parametrization (*p*_T_>5 GeV/*c*) described in the text. *Bottom*: Ratio of data to parametrization. The *gray band* indicates the total *p*_T_ dependent systematic uncertainty of the data, *open circles* show data points only used for the evaluation of the systematic uncertainty of the parametrization (Color figure online)

Figure 3 shows the differential cross section in INEL pp collisions as a function of *p*_T_ for $\sqrt{s} = 2.76~\mbox{TeV}$ together with the parametrization for *p*_T_>5 GeV/*c*. The ratio between data and parametrization in the lower panel demonstrates the good agreement of the parametrization with the data. The gray band indicates the total *p*_T_ dependent systematic uncertainty of the measured spectrum as presented in Table 1.

To estimate the systematic uncertainty of the parametrization and extrapolation, the lower boundary of the fit range of the Hagedorn parametrization is varied between *p*_T_=3 GeV/*c* and *p*_T_=7 GeV/*c*, while the upper boundary is fixed to the highest data point measured at *p*_T_=32 GeV/*c*. Together with the systematic uncertainties on the measured differential cross section as shown in Table 1 this results in a total systematic uncertainty on the reference at $\sqrt{s} = 2.76\ \mbox{TeV}$ of 6.4 % for low *p*_T_ up to 19 % at *p*_T_=50 GeV/*c*.

The final pp reference for the determination of *R*_AA_ at $\sqrt{s} = 2.76\ \mbox{TeV}$ is constructed from the measured data points up to *p*_T_=5 GeV/*c* and the parametrization for *p*_T_>5 GeV/*c*. Statistical uncertainties in the extrapolated part of the reference are obtained from the covariance matrix of the parametrization. The systematic uncertainties on the spectrum are propagated to the reference by application of the full extrapolation procedure using the measured data points shifted up and down by the total systematic uncertainty.

**Fig. 4:**
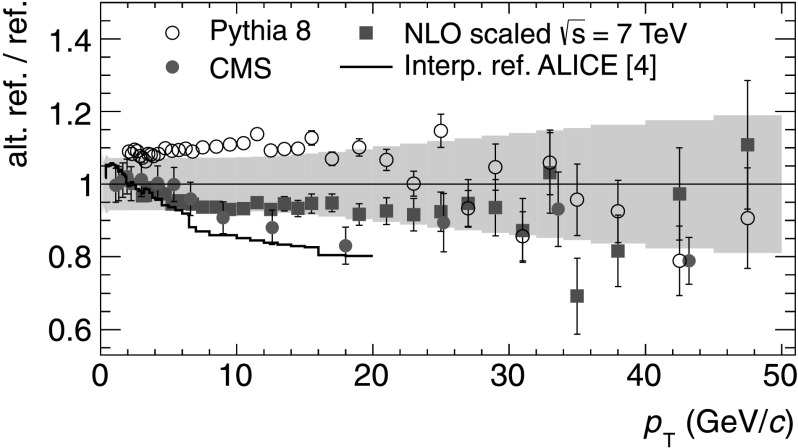
Ratio of alternative references to the new constructed pp reference at $\sqrt{s} = 2.76\ \mbox{TeV}$ as discussed in the text. The *gray band* indicates the total *p*_T_ dependent systematic uncertainty as discussed in the text. The overall normalization systematic uncertainties ±1.9 % (±6 %) for ALICE (CMS) are not shown (Color figure online)

This reference is compared to alternative measurements and approaches. Figure 4 shows the ratio between alternative pp references and the reference at $\sqrt{s} = 2.76\ \mbox{TeV}$ presented in this paper. Above *p*_T_=20 GeV/*c*, all references agree within the systematic uncertainties. Simulations with the PYTHIA8 generator [17] agree with the new reference for *p*_T_>15 GeV/*c*. Below *p*_T_=20 GeV/*c*, the shape of the PYTHIA8 spectrum is similar to the measured reference. A pp reference presented by the CMS collaboration [18] agrees best for *p*_T_<6 GeV/*c*. The overall normalization systematic uncertainties ±1.9 % (±6 %) for ALICE (CMS) are not included in the comparison. A reference based on an interpolation between measured yields at $\sqrt {s} = 0.9\ \text{and}\ 7~\text{TeV}$ as discussed in [6] does not agree with the new reference for *p*_T_>6 GeV/*c*. Finally a scaling of the measured differential cross section in INEL pp collisions at $\sqrt{s} = 7\ \mbox{TeV}$ with the ratio of pQCD calculations (as shown in Fig. 2)

3 $$\begin{aligned} {\mathrm{d}}^2 \sigma_{\mathrm{ch}}^{\mathrm{pp}} / {\mathrm{d}} \eta\,{\mathrm{d}}p_{\mathrm{T}} |_{2.76\ \mathrm{TeV}} & = \frac{ \mathrm{d}^2 \sigma_{\mathrm{ch}}^{\mathrm{pp}} / {\mathrm{d}} \eta\,{\mathrm{d}}p_{\mathrm{T}}| _{\mathrm{NLO}, 2.76\ \mathrm{TeV}} }{ \mathrm{d}^2 \sigma_{\mathrm{ch}}^{\mathrm{pp}} / {\mathrm{d}} \eta\,{\mathrm{d}}p_{\mathrm{T}} |_{\mathrm{NLO},\ 7\ \mathrm{TeV}} } \\ &\quad \times \mathrm{d}^2 \sigma_{\mathrm{ch}}^{\mathrm{pp}} / {\mathrm{d}} \eta\,{ \mathrm{d}}p_{\mathrm{T}}|_{7\ \mathrm{TeV}} \end{aligned}$$

agrees well in shape and normalization with the measured data over a wide range in *p*_T_. The systematic uncertainty of the new reference is indicated in Fig. 4 as a gray band for comparison.

## Construction of a pp reference for $\sqrt{s} = 5.02\ \mbox{TeV}$

Similar to *R*_AA_, a nuclear modification factor *R*_pA_ in proton-lead collisions has been studied [19] at $\sqrt{s} = 5.02\ \mbox{TeV}$. No measured pp reference is available at this collision energy. Due to the asymmetric p–Pb collision system, the *η* coverage of the detector is shifted with respect to the symmetric pp or Pb–Pb collisions. To obtain a maximum overlap between the pp and p–Pb systems, a pp reference is needed for |*η*|<0.3. To construct the pp reference at this energy, different methods for three *p*_T_-ranges are combined.

0.15<*p*_T_<5 GeV/*c*: As NLO-pQCD becomes unreliable for small *p*_T_, the measured differential cross sections for pp collisions of $\sqrt{s} = 2.76\ \mbox{and}\ 7~\text{TeV}$ are interpolated for a given *p*_T_, assuming a power-law behavior of the $\sqrt{s}$ dependence of the cross section. Here the maximum relative systematic uncertainty of the underlying measurements has been assigned as systematic uncertainty.

5<*p*_T_<20 GeV/*c*: The measured differential cross section for pp collisions at $\sqrt{s} = 7\ \mbox{TeV}$ is scaled to $\sqrt{s} = 5.02\ \mbox{TeV}$ using the NLO-pQCD calculations (Eq. (3)). Systematic uncertainties are determined by taking into account differences to an interpolated reference as well as to a scaled reference using *μ*=*p*_T_/2 and *μ*=2*p*_T_ as alternative choices for the renormalization and factorization scales.

*p*_T_>20 GeV/*c*: The NLO-scaled reference is parametrized in the range 20<*p*_T_<50 GeV/*c* by a power-law function and the parametrization is used.

**Fig. 5:**
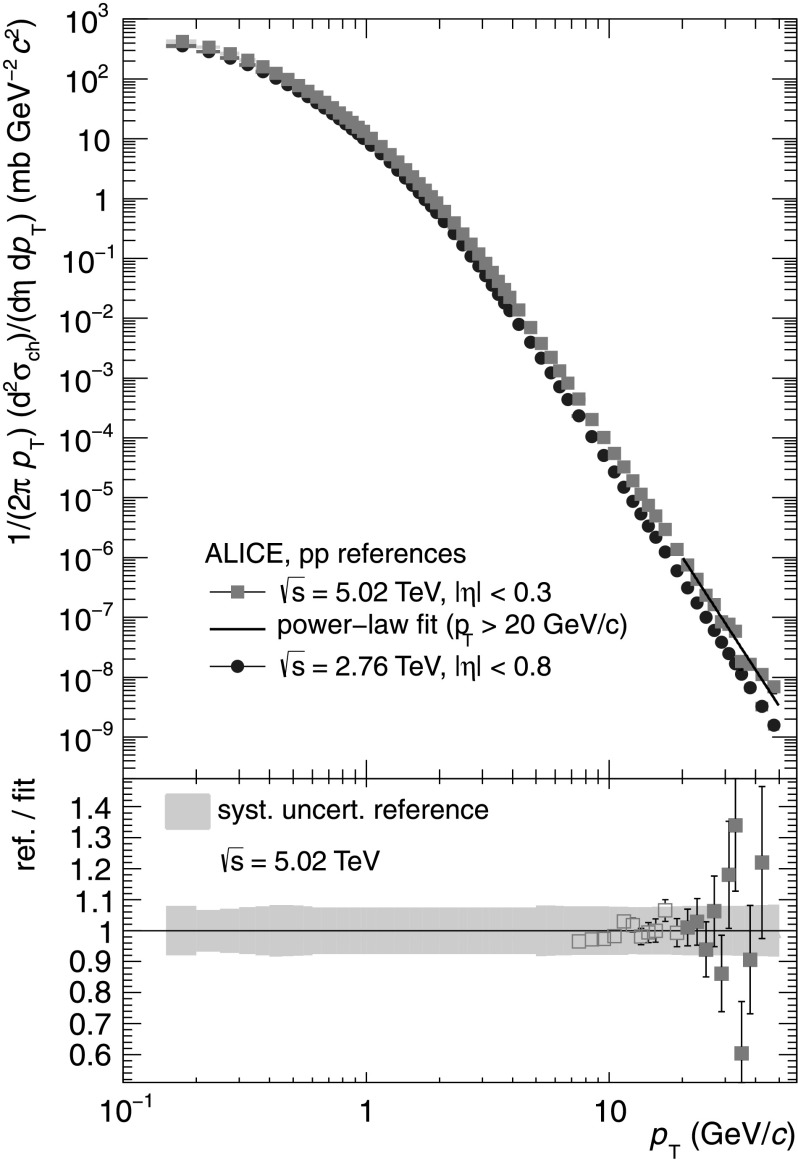
*Top*: Constructed pp references for $\sqrt{s} = 2.76\ \text{and} \ \sqrt{s} = 5.02\ \mbox{TeV}$. *Bottom*: Comparison of NLO-scaled reference and parametrization. The parametrization is used for *p*_T_>20 GeV/*c*. The *gray band* indicates the total *p*_T_ dependent systematic uncertainty as discussed in the text (Color figure online)

The constructed pp reference for $\sqrt{s} = 5.02\ \mbox{TeV}$ is shown in Fig. 5 together with the reference for $\sqrt{s}=2.76~\mbox{TeV}$ discussed above. For *p*_T_>20 GeV/*c* the data points show the NLO-scaled reference which is parametrized by a power-law function (line) to obtain the final reference at $\sqrt {s} = 5.02\ \mbox{TeV}$. In the bottom part of the figure a comparison of the NLO-scaled reference and the parametrization is shown.

## Summary

Differential cross sections of charged particles in inelastic pp collisions as a function of *p*_T_ have been presented for $\sqrt{s} = 0.9,\ 2.76\ \text{and}\ 7~\text{TeV}$. Comparisons of the *p*_T_ spectra with NLO-pQCD calculations show that the cross section for an individual value of $\sqrt{s}$ cannot be described by the calculation. The relative increase of cross section with $\sqrt{s}$ is well described by NLO-pQCD, however. The systematic comparison of the energy dependence can help to tune the model dependent ingredients in the calculation. Utilizing these observations and measurements procedures are discussed to construct pp reference spectra at $\sqrt{s} = 2.76$ (|*η*|<0.8) and 5.02 TeV (|*η*|<0.3) in the corresponding *p*_T_ range of charged particle *p*_T_ spectra in Pb–Pb and p–Pb collisions measured by the ALICE experiment. The reference spectra are used for the calculation of the nuclear modification factors *R*_AA_ [10] and *R*_pA_ [19]. The systematic uncertainties related to the pp reference were significantly reduced with respect to the previous measurement by using the *p*_T_ distribution measured in pp collisions at $\sqrt{s}=2.76~\mbox{TeV}$.
